# Primary Thyroid Stimulating Hormone Screening for Congenital Hypothyroidism in King Abdullah Hospital, Bisha, Saudi Arabia

**DOI:** 10.7759/cureus.7166

**Published:** 2020-03-02

**Authors:** Mohammed Abbas, Eltayeb Tayrab, Abedelmonium Elmakki, Jowayria Tayrab, Abdullah Al-shahrani, Elhadi Miskeen, Karimeldin Salih

**Affiliations:** 1 Pediatrics, College of Medicine, University of Bisha, Bisha, SAU; 2 Chemical Pathology, Faculty of Medical Laboratory Sciences, National Ribat University, Khartoum, SDN; 3 Medical Laboratory Sciences, Faculty of Applied Medical Sciences, University of Bisha, Bisha, SAU; 4 Pediatrics, King Abdullah Hospital, Bisha, SAU; 5 Family Medicine, College of Medicine, University of Bisha, Bisha, SAU; 6 Obstetrics and Gynecology, College of Medicine, University of Bisha, Bisha, SAU; 7 Pediatrics/Medical Education, College of Medicine, University of Bisha, Bisha, SAU

**Keywords:** thyroid stimulating hormone, neonatal screening, congenital hypothyroidism, cord blood, bisha, saudi arabia

## Abstract

Background

Thyroid stimulating hormone (TSH) concentration in cord blood measured at birth has been proposed as an indicator of congenital hypothyroidism (CH). Elevated TSH levels at birth were associated with cognitive and psychomotor disorders among young children.

Objectives

The purpose of this study is to investigate the epidemiology of CH using cord blood TSH screening among 2501 newborn infants in the period from January to November 2016 in Bisha Province, Saudi Arabia.

Methodology

This cross-sectional, hospital-based study was conducted at King Abdullah Hospital, Bisha, which is a secondary care referral district general hospital with 400 beds. All neonates born in the designed period were included and screened according to the standard CH screening protocol. Data was collected from all neonates born in the designed period. The screening was performed according to the standard CH screening protocol. After birth, cord blood specimens were used. TSH was measured by Perkin Elmer ELISA machine. The data were analyzed using the Statistical Package for the Social Sciences (SPSS) version 24.0 (IBM Corp., Armonk, NY). A p-value of less than or equal to 0.05 was considered significant.

Main results

A total of 1308 (52.3%) neonates were males, while 1193 (47.7%) were females. Serum TSH levels were ranged from 0.01-73.9 µU/ml. The mean ± SD was (7.60 ± 6.02 µU/ml); the cutoff point was 25 µU/ml.

Conclusion

The prevalence of congenital hypothyroidism among Saudi neonates in Bisha Province is 1:834, which is the highest in the rejoin. The prevalence of hypothyroidism from the total screened population was (0.12%). We recommended screening with special consideration to those with high TSH in the primary screening.

## Introduction

Congenital hypothyroidism (CH) is the most common pediatric endocrine disorder [1,2]. CH is sporadic and occurs in 1:1500 to 1:4000 newborns, making it one of the most common causes of preventable mental retardation [3-5]. CH is less prevalent in Blacks and more prevalent in Caucasians [6]. Unrecognized CH leads to cognitive disorders [7]. CH delays psychomotor development in infants [5,8]. Neonatal screening programs for CH allow early suitable diagnosis and treatment of the condition [4]. Screening programs are very important clinically because severe cases without prompt treatment will lead to irreversible mental retardation [1,5,9]. Primary screening of thyroid-stimulating hormone (TSH) levels has become standard in many parts of the world [7]. If the diagnosis of hypothyroidism is made a few weeks after birth and the treatment starts early, neurodevelopmental outcomes are generally normal [10,11]. The first screening program for CH was conducted in North America in 1972 [4]. There are many diseases and conditions present with increased TSH levels; these include CH [5]. The majority of newborns with CH do not have clear manifestations of hypothyroidism, making clinical diagnosis difficult [10]. TSH is the key indicator in CH screening [12]. CH screening uses (TSH) and/or thyroxine (T4) as indicators [13]. TSH is synthesized and secreted in the pituitary gland in response to Thyrotropin-releasing hormone (TRH) from the hypothalamus [2]; it is secreted in the blood in a pulsatile manner, with a mean pulse amplitude of 0.6 mU/L and a frequency of 5 to 20 pulses per 24 hours [14]. TSH concentration in the whole blood measured at birth has been proposed as an indicator of iodine status [15]. Neonatal TSH is also useful in detecting severe iodine deficiency [16]. Most infants with CH are normal at birth and show no signs, emphasizing the importance of TSH screening for early detection of CH [9]. Elevated TSH levels at birth were associated with suboptimal cognitive and psychomotor outcomes in children [15]. In most cases, mothers with CH pass the disease to their children [16,17]. The goal of early diagnosis and initial therapy of CH is to minimize the exposure of the neonatal central nervous system to hypothyroidism by normalizing thyroid function as rapidly as possible [9]; it is also considered a monitoring tool in programs of iodine supplementation [18]. Hence, an optimal cutoff level of TSH in screening is critical to ensuring that true cases of CH are not missed [19]. Disorders involving iodide transport, including CH, affect individuals during their whole lifespan and, if not diagnosed or improperly treated, can have a profound impact on child growth, metabolism, cognitive development, and quality of life [2].

The purpose of this study was to investigate the epidemiology and prevalence of CH using TSH screening among newborns in Bisha Province, Saudi Arabia.

## Materials and methods

This cross-sectional, hospital-based study was conducted at King Abdullah Hospital, Bisha, which is a secondary care referral, general district hospital with 400 beds, serving 500,000 people in Southern Saudi Arabia. In this study, we used the data collected for CH screening from 2501 neonates in Bisha Province in the period from January 2016 to November 2016. All neonates born in the designed period were included in the study without any exclusions; the screening was done according to the standard CH screening protocol. Cord blood specimens (2 ml) were used after birth. Serum TSH was measured by a PerkinElmer ELISA machine. The kit used was also from the PerkinElmer Company (Waltham, MA). The internal controls (1, 2, and 3) were used with a range of 0.4 to 32.6 µU/ml, while a Bio-Rad control system was used for external quality control samples. The screening was done according to the national screening program protocol. For suspicious results, TSH values were rechecked, and a clinical follow-up with thyroid-function tests was conducted. The results were passed to the pediatric department for prompt consideration, and if hypothyroidism was confirmed, treatment was started using a single dose of levothyroxine (LT4; 10-15 μg/kg/day) as per the guidelines of the American Academy of Pediatrics [7]. If the initial TSH level was ≥25 µU/ml, treatment was suggested, and thyroid-function tests were performed concomitantly. The cutoff point of TSH was 25 µU/ml. For results that exceeded 40 µU/ml or were suspicious, the serum TSH levels were rechecked to confirm or exclude CH.

The data were analyzed using the Statistical Package for the Social Sciences (SPSS) version 24.0 (IBM Corp., Armonk, NY). A p-value of less than or equal to 0.05 was considered significant. The results are displayed in tables and figures.

Ethical consideration

Informed written consent was obtained from the fathers or mothers of the neonates after they agreed to participate in this study.

Statistical analysis

As mentioned, the data were analyzed using SPSS version 24. The frequency and the mean and standard deviation (SD) for the demographic data and TSH levels in the neonates were calculated.

## Results

This study, which included 2501 neonates, revealed that 1308 (52.3%) of the neonates were males, while 1193 (47.7%) were females. The study also showed that 2308 (92.3%) of the newborn babies involved in the research were Saudi natives, while 187 (7.5%) were non-Saudi; the nationalities of six (0.24%) neonates were unknown. Regarding socioeconomic class, 1580 (63.2%) participants were middle class, while 921 (36.8%) were high class (Table 1).

**Table 1:** The descriptive study of neonatal TSH concentrations (µU/ml) in King Abdullah Hospital, Bisha, KSA (N = 2501). TSH: Thyroid stimulating hormone

Distribution of TSH level in the study (µU/ml)	No. of neonates	Percentage (%)
<5	1030	41.18%
5<10	941	37.62%
10<15	283	11.32%
15<20	127	5.08%
20<25	57	2.28%
25<30	35	1.40%
30<35	13	0.52%
35<40	11	0.44%
40<45	1	0.04%
45<50	0	0.0
>50	3	0.12%
Total	2501	100%

Cord blood serum TSH levels ranged from 0.01 to 73.9 µU/ml. The mean ± SD was 7.60 ± 6.02 µU/ml. The distribution of TSH levels among the neonates is shown in Table 2. The cutoff point for TSH in the method used was 25 µU/ml. The TSH levels exceeded the cutoff point were less than 40 µU/ml in 63 cases (2.5%). For four (0.16%) neonates, the TSH levels were higher than 40 µU/ml. Values higher than 50 µU/ml and confirmed after rechecking were found in three (0.12%) neonates. The overall prevalence of hypothyroidism in Saudi infants in Bisha Province is 1:834.

**Table 2:** Distribution of the study population according to general characteristics (N = 2501).

General characteristics	Frequency	Percentage (%)	P value
Sex			
Male	1308	52.3	0.60
Female	1193	47.7	
Nationality			
Saudi	2308	92.3	0.816
Non-Saudi	187	7.5	
Missed data	6	0.24	
Socioeconomic status			
Moderate	1580	63.2	0.5
High	921	36.8	
Total	2501	100	

**Figure 1:**
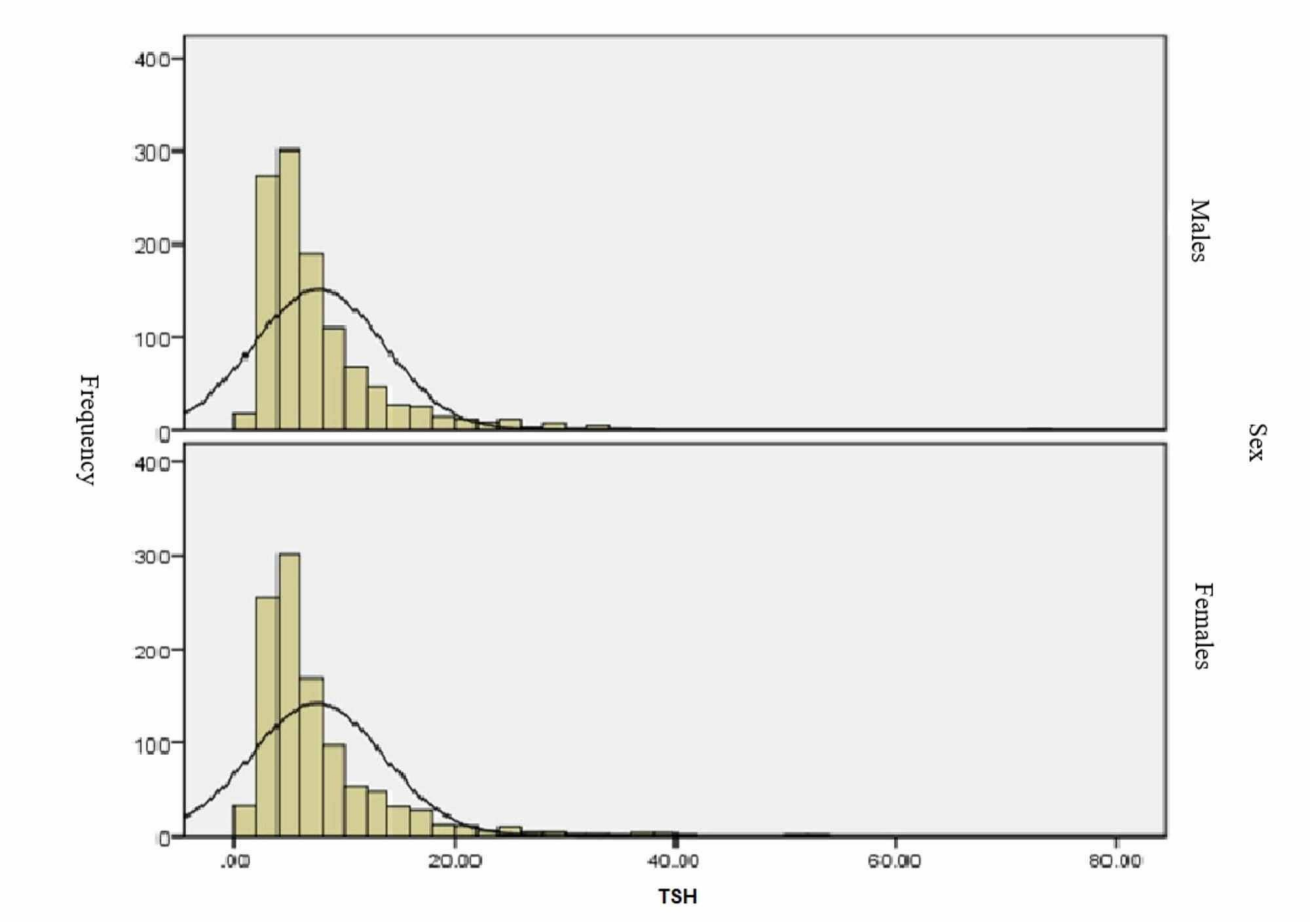
Distribution of neonatal thyroid stimulating hormone (TSH) concentrations (µU/ml) according to the standard TSH categorization in King Abdullah Hospital, Bisha, Kingdom of Saudi Arabia (N = 2501).

The distribution of neonatal TSH concentrations (µU/ml) by gender was skewed to the right, with no variation in gender distribution. The associations were tested with a chi-square test, which showed no statistically significant differences according to gender (p = 0.60), blood group (p = 0.623), or nationality (p = 0.816) (Figure 1).

**Figure 2:**
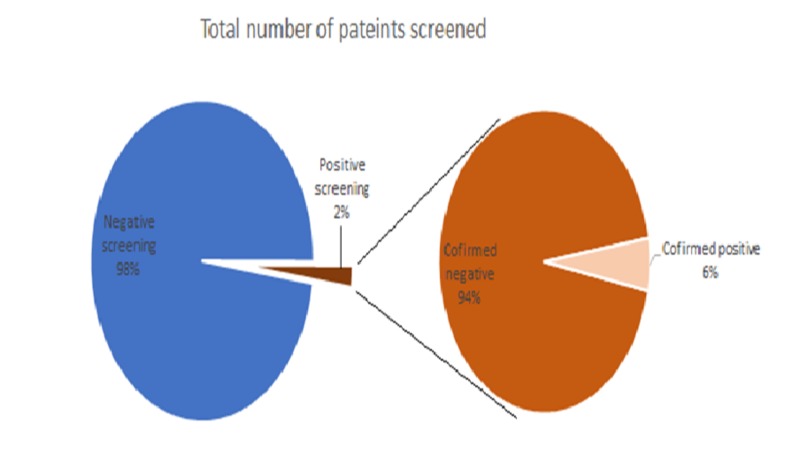
Distribution of neonatal thyroid stimulating hormone (TSH) concentrations (µU/ml) according to the further assessment of the positive hypothyroidism cases.

Further screening of positive findings (values higher than 25 µU/ml) was carried out by a comprehensive clinical laboratory assessment and close follow-up. According to the King Abdullah Hospital protocol, the laboratory confirmation followed a comprehensive multidisciplinary assessment as per the guidelines of the American Academy of Pediatrics [7]. It was found that only three (5.5%) out of 54 cases were confirmed to have CH. The prevalence of hypothyroidism was calculated from the total screened population and was found to be 3:2501 (0.12%) (Figure 2).

## Discussion

CH is the most common pediatric endocrine disease, which threatens early infancy with permanent mental retardation if not diagnosed early and treated. This research assessed CH using cord blood TSH because this technique has become the gold standard in many parts of the world, as reported by the American Academy of Pediatrics, and because TSH screening is more specific in the diagnosis of CH [7,9]. It is known that in civilized countries, neonatal screening is widely adopted, which enables early diagnosis of CH in neonates, so severe mental retardation due to CH is very rare in these countries [11]. On the other hand, we adopted this technique and not another one because hypothyroidism assessed by capillary TSH needs to be confirmed by venous TSH [1]. In this study, we directly used cord blood samples because TSH testing programs that use peripheral blood need serial screening at two and six weeks of age; this is difficult in Bisha for many reasons, one of which is that the early discharge of mothers postpartum increases the proportion of false-positive TSH elevations. With the failure of repeated testing in the peripheral blood sampling method, approximately half of the newborns with congenital thyroid hormone deficiency will be missed [20,21]. It is well known that in Saudi Arabia, the TSH screening program for CH was started earlier in 1989 [22]. When initiated, the screening program in Saudi Arabia used cord serum TSH as an indicator of CH. In this study, the overall prevalence of hypothyroidism in Saudi infants in Bisha Province, Southern Saudi Arabia, is 1:834. This finding is in disagreement with other local studies [23]; in Northern Saudi Arabia, in Madina Munawara, the prevalence was 1:4000, which is lower than the prevalence obtained in the present study. The present study also found a higher prevalence than another local study [22]. Furthermore, the prevalence found in our study is also higher than that reported in several international studies [3-5]. This unexplainable result raises many questions. The higher prevalence in the current study may be due to a physiological TSH surge in the early hours after delivery when we collected the cord blood [9,24]. In addition, the particular population of this study, in which all neonates born in the designed period were included, may have resulted in false-positive cases in immature infants and infants with a low birth weight [9]. Alternatively, if the confounding environmental or demographic factors had no effect, CH prevalence in Bisha in Southern Saudi Arabia would be the highest in the region. This will need another study with a big sample size to prove or disprove the finding of this research.

In the present study, only four neonates had TSH values of more than 40 µU/ml, which were borderline, and three neonates had values of more than 50 µU/ml, as shown in Table 1. All the suspicious results and those with values of more than 50 µU/ml were repeated. After the laboratory results were rechecked, three neonates were confirmed to have CH, and for them treatment was started with a single dose of levothyroxine (LT4; 10-15 μg/kg/day); then, regular follow-up was planned.

On the other hand, one of the neonatal blood samples had a very low value of 0.01 µU/ml; unfortunately, we missed the patient when we tried to follow him. This may be due to secondary causes of pituitary disorders.

In this study, the pediatricians at King Abdullah Hospital maintained clinical vigilance of the neonates during the research. Thyroid hormone deficiency at birth is most commonly caused by thyroid gland maldevelopment (dysgenesis) or biosynthesis defects (dyshormonogenesis). These disorders result in primary, secondary, or central hypothyroidism at birth, causing TSH deficiency [3].

Finally, the measurement at birth of the TSH concentration in the whole blood has many other purposes. For example, it has been proposed as an indicator of maternal iodine status [15].

## Conclusions

One of the best and fruitful screening programs in Kingdom of Saudi Arabia is neonatal screening program for the TSH, where umbilical blood sample is usually taken for that purpose. According to our research findings, the prevalence of hypothyroidism is the highest in the region. Nationwide studies should be performed for direct assessment and monitoring of TSH status in vulnerable populations to confirm the accuracy of these results.
